# LIMITED FUTURE TIME PERSPECTIVE IS ASSOCIATED WITH LOWER EMOTIONAL WELL-BEING DURING THE COVID-19 PANDEMIC

**DOI:** 10.1093/geroni/igac059.752

**Published:** 2022-12-20

**Authors:** Yochai Shavit, Jessica Barnes, Laura Carstensen

**Affiliations:** Stanford Center on Longevity, Stanford University, Stanford, California, United States; Stanford University, Stanford, California, United States; Stanford University, Stanford, California, United States

## Abstract

Socioemotional selectivity theory postulates that limited future time perspective (FTP) motivates older adults to prioritize emotionally meaningful goals, explaining documented age advantages in emotional well-being. During the early months of the COVID-19 pandemic, we collected data from 945 community dwelling adults and 156 assisted living facilities residents living in the United States (N= 1101, age-range: 18-98). Participants reported their FTP using the scale developed by Carstensen and Lang (1996), as well as the frequency and intensity of sixteen positive and thirteen negative emotions. Age association with limited FTP was comparable to past studies. Contrary to our hypotheses, limited FTP was associated with lower emotional well-being across ages and suppressed (rather than mediated) a general trend towards higher emotional well-being in older ages. Findings suggest that there may be conditions under which perceptions of limited time horizons have negative implications. Theoretical implications are discussed.

